# 14.1 VIOLENT CRIME IN SCHIZOPHRENIA AND BIPOLAR DISORDER: A POPULATION-BASED STUDY

**DOI:** 10.1093/schbul/sby014.054

**Published:** 2018-04-01

**Authors:** Anat Fleischman, Nomi Werbeloff, Rinat Yoffe, Michael Davidson, Mark Weiser

**Affiliations:** 1 Sackler Faculty of Medicine, Tel-Aviv University; 2 Sheba Medical Center; 3 Division of Mental Health Services, Ministry of Health, Jerusalem

## Abstract

**Background:**

Previous studies have found that patients with schizophrenia and bipolar disorder are more likely to be violent than the general population. The aim of this study was to investigate the association between schizophrenia and bipolar disorder and violent crime in the Israeli population.

**Methods:**

Using the Israeli Psychiatric Hospitalization Case Registry we identified 3187 patients with a discharge diagnosis of schizophrenia and 506 patients with a discharge diagnosis of bipolar disorder. For each proband we identified parents and siblings, and gender- and age-matched controls for patients, parents and siblings. Information on violent crimes was obtained from police records.

**Results:**

Patients with schizophrenia were at increased risk for violent crimes compared with controls [odds ratio (OR) 4.3, 95% confidence interval (CI) 3.8–4.9], especially women (OR 9.9, 95% CI 6.2–15.7). Risk for violent crimes was higher among patients with co-morbid substance misuse than in patients without such co-morbidity (OR 5.1, 95% CI 4.2–6.3).

Patients with diagnosis of bipolar disorder were 2.5 times more likely to be convicted or released for mental reasons of violent crimes compared with controls and unaffected full siblings (OR=2.5, 95%CI 1.7–3.7, OR=2.5, 95%CI 1.6–4.0 respectively). Although men were more violent than women, diagnosis of bipolar disorder was a more significant risk factor for female patients than for male patients (OR=16.1 95%CI 1.8–144.6 vs. OR=2.4, 95%CI 1.5–3.7).

**Discussion:**

The results of this study suggest that increased risk of violence is part of the clinical picture of schizophrenia and bipolar disorder and needs to be recognized as a legitimate, essential, aspect of clinical management.

